# Contrasting PTH Response of Denosumab Use in Dialysis Patients: A Report of 2 Cases

**DOI:** 10.3390/pharmacy8020059

**Published:** 2020-04-01

**Authors:** Soo Min Jang, Smitha Anam, Tara Pringle, Paul Lahren, Sergio Infante

**Affiliations:** 1 Department of Pharmacy Practice, Loma Linda University School of Pharmacy, Loma Linda, CA 92350, USA; SmJang@llu.edu; 2 Loma Linda University Kidney Center, Loma Linda, CA 92354, USA; TPringle@llu.edu (T.P.); PLahren@llu.edu (P.L.); 3 Pacific Nephrology, Concord, CA 94520, USA; Smitha.Anam_MD@Johnmuirhealth.com; 4 Department of Internal Medicine Nephrology Division, Loma Linda University School of Medicine, Loma Linda, CA 92350, USA

**Keywords:** denosumab, calcium, parathyroid hormone, end-stage renal disease, hemodialysis

## Abstract

A common complication of end-stage renal disease (ESRD) is mineral and bone disorder. Yet, many anti-osteoporotic drugs are contraindicated in ESRD patients. Denosumab, a monoclonal antibody, does not require renal dose adjustment. However, its use is uncertain due to a lack of safety and efficacy of data in this population. Two hemodialysis patient cases of contrasting responses in parathyroid hormone (PTH) after denosumab administration were observed. Patient 1, a 62-years-old male received denosumab 60 mg at Day 0. His calcium decreased from 8.8 mg/dL to 6.8 mg/dL on Day 30. The PTH level increased from 265 pg/mL to 372 pg/mL after 30 days. Calcium and PTH levels approached normal range after increasing doses of vitamin D/calcium supplements, and calcitriol. Patient 2, a 72-years-old male on hemodialysis also received denosumab 60 mg on Day 0. His baseline calcium and PTH were 9.2 mg/dL and 420 pg/mL, respectively. On Day 30, his calcium level decreased (6.8 mg/dL) but, PTH level drastically increased (>5000 pg/mL). Denosumab commonly causes hypocalcemia and hyperparathyroidism since it inhibits osteoclast activation, reduces calcium release from bone and increases PTH levels as a compensatory mechanism. With a wait-and-watch approach, Patient 2’s levels approached the normal range (calcium 9.6 mg/dL and PTH 274 pg/mL at Day 90).

## 1. Introduction

Mineral and bone metabolism disorders are common in end-stage renal disease (ESRD) patients [1]. ESRD and dialysis disturb tightly regulated calcium (Ca), intact parathyroid hormone (iPTH) and vitamin D (VitD) metabolisms. These lead to bone density reduction, metabolic bone diseases, a higher prevalence of fractures, and a higher mortality rate after the fracture in advanced chronic kidney disease (CKD) patients [1,2]. Moreover, osteoporosis management is challenging in ESRD patients because treatments such as bisphosphonates are contraindicated in ESRD patients. 

Denosumab is a human monoclonal antibody directed against the receptor activator of nuclear factor kappa B ligand (RANKL). This reduces the formation, function, and survival of osteoclasts [3]. The U.S. Food and Drug Administration approved denosumab as an antiresorptive therapy in patients with osteoporosis and renal impairment [4]. Denosumab-associated severe hypocalcemia is a common adverse effect in the general population as well as patients with advanced CKD [5,6,7,8,9]. As a result, an increase in iPTH level is also commonly seen in order to compensate for a decrease in calcium [10,11]. However, changes in iPTH level from denosumab use in ESRD patients have been conflicting in previous reports [6,7,11]. We present two ESRD patients receiving hemodialysis who experienced asymptomatic hypocalcemia and contrasting iPTH responses after denosumab treatment for osteoporosis.

## 2. Case Report

These patients had similar demographic characteristics (sex, race, 25-hydroxy vitamin D, Ca level, iPTH level, Ca bath, and calcimimetic use (Table 1)). Patient’s age (62 years-old vs. 72 years-old), dialysis treatment vintage (2 years vs. 11 years), and use of denosumab and calcitriol were different. Calcitriol regimen for Patient 1 was calcitriol 0.25 mcg orally three times weekly whereas, Patient 2 administered calcitriol 0.25 mcg orally daily. Patient 1 used alendronate for two years (no Ca/VitD supplement use in our electronic health records) and stopped it a year prior to denosumab use. Patient 2 never used bisphosphonate before but was on Ca 1000 mg-VitD_3_ 400 mg daily.

**Figure 1:**
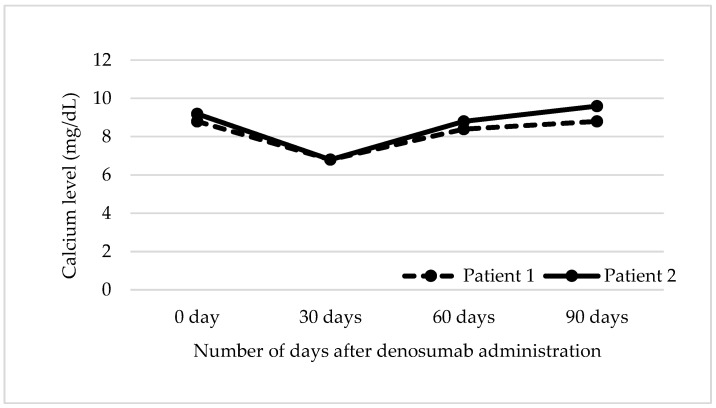
Calcium Results over 90 days. Similar changes were observed in calcium levels between Patient 1 and Patient 2. Calcium levels (mg/dL) for Patient 1 were: 8.8 (0 day), 6.8 (30 days), 8.4 (60 days) and 8.8 (90 days). For Patient 2, calcium levels were: 9.2 (0 day), 6.8 (30 days), 8.8 (60 days) and 9.6 (90 days).

**Figure 2:**
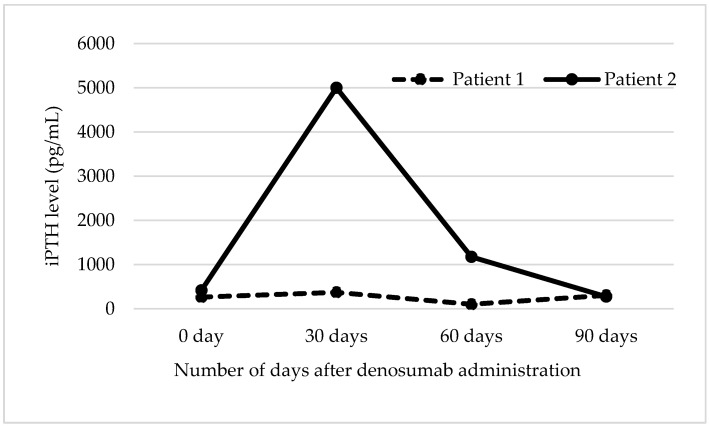
Intact Parathyroid Hormone Results over 90 days. Intact parathyroid hormone (iPTH) levels (pg/mL) for Patient 1 were: 265 (0 day), 372 (30 days), 103 (60 days) and 303 (90 days). For Patient 2, the levels were: 420 (0 day), >5000 (30 days), 1173 (60 days) and 274 (90 days). Patient 1 and Patient 2 had contrasting responses in iPTH at Day 30.

Baseline and repeated Ca and iPTH levels at 0 days, 30 days, 60 days, and 90 days after denosumab administrations are shown in Figure 1 and Figure 2, respectively. Both patients experienced an asymptomatic hypocalcemia (6.8 mg/dL) at Day 30. Patient 1 had a slight increase in iPTH level (from 265 pg/mL to 372 pg/mL) as a compensatory response. Subsequently, calcitriol dose was increased from 0.25 mcg three times weekly to 1 mcg daily, VitD_3_ 2000 mg daily and Ca 600 mg daily. His Ca levels (albumin level of 4 g/dL) were 8.4 mg/dL at Day 60 and 8.8 mg/dL at Day 90. In addition, iPTH levels changed to 103 pg/mL (Day 60) and 303 pg/mL (Day 90). In striking contrast, a drastic increase in iPTH level was observed in Patient 2 (despite being on Ca 1000 mg-VitD_3_ 400 mg and calcitriol 0.25 mcg daily) to 4178 pg/mL as a compensatory response. Confirmatory re-draw for iPTH level was repeated after three days, and the result was >5000 pg/mL. With no additional intervention, Patient 2’s corrected Ca level (albumin level of 3.1 g/dL) trended towards the normal range by Day 90 (Figure 2). DEXA scans were not repeated since 2018 for both patients (electronic health record was checked in 2020).

## 3. Discussion

To our knowledge, this is the first case report that showed a significant transient iPTH increase (>5000 pg/mL) from denosumab use in patients receiving maintenance hemodialysis. Denosumab is an anti-resorptvie medication that inhibits early stages of osteoclast maturation [12]. It is well-known to induce a dose-dependent iPTH increase as a compensatory mechanism against decreases in calcium levels [13]. Previous reports showed that iPTH increase (2- to 3-fold) occurs within the first 30 days of denosumab administration. Yet, the increase in iPTH level can be observed up to 6 months [13,14].

Block et al. performed a single-dose study of denosumab in patients with various degrees of kidney disease. Among eight dialysis-dependent patients that were included in the study, two patients had serum Ca level of <7.5 mg/dL. The average iPTH level was 158.2 ± 125.1; however, the study excluded ESRD patients with iPTH ≥300 pg/mL [7]. McCormick et al. also reported the case of a female hemodialysis patient who experienced severe hypocalcemia (total serum Ca 5.4 mg/dL) and increased iPTH levels (baseline, 186 pg/mL to 1044 pg/mL) 30 days after receiving a single denosumab injection [15]. The patient reported a fatigue, which started 2 weeks post-injection and continued until calcium level was normalized (50 days post-injection). Patient’s dialysate calcium bath was increased from 2.5 to 3.5 mEq/L and calcitriol 1 mcg daily treatment was initiated for hypocalcemia [15]. A retrospective study by Festuccia et al. showed improvement in bone metabolism with denosumab in 12 hemodialysis patients without significant safety concerns over 24 months [8]. They observed a decrease in average serum Ca: 9 mg/dL at baseline, 8.1 mg/dL at 3 months, 9.4 mg/dL at 6 months, 9 mg/dL at 9 months, 9.8 mg/dL at 12 months, 8.9 mg/dL at 15 months, 9.2 mg/dL at 18 months, 9.3 mg/dL at 21 months, and 9.1 mg/dL at 24 months. The average iPTH levels were: 656 pg/mL at baseline, 1359 pg/mL at 3 months, 530 pg/mL at 6 months and the highest iPTH of 1576 pg/mL at 9 months. 

Chen et al. conducted a 24-week open-label study in Taiwan to evaluate the short-term effect of the co-administration of calcitriol and denosumab on severe secondary hyperparathyroidism in dialysis patients [10,11]. Three patients experienced paresthesia and myalgia from hypocalcemia (serum Ca range 5.5–6.9 mg/dL), but none of them required hospitalization [10]. They showed a significant decrease in iPTH (mean decrease, 58 ± 6%, *P* < 0.01) with denosumab/calcitriol administration compared to the control group (no denosumab). Parathyroid gland volume was decreased (mean decrease, 22 ± 5%) with denosumab/calcitriol administration (*P* < 0.01) and the gland volume progressively increased (21 ± 5%) in the control group (*P* < 0.05). Although a third of the cohort resulted in hypocalcemia, it was rapidly resolved with calcium and calcitriol supplements. The authors concluded that denosumab allows for supra-physiologic doses of calcitriol resulting in decreased parathyroid secretion and parathyroid hyperplasia. 

Hiramatsu and colleagues conducted a prospective non-controlled study with 11 Japanese hemodialysis patients with a single dose of denosumab [16]. They reported asymptomatic hypocalcemia with nadir Ca range of 6.8–8.9 mg/dL as well as a compensatory iPTH increase (range of 159–1938 pg/mL at Day 30). Serum Ca and iPTH returned to baseline levels by 6 months with active VitD and calcium supplementation. Takami et al. presented a case-control study comparing Japanese hemodialysis patients who received denosumab (*n* = 17) versus not receiving denosumab (*n* = 20) and showed an increase in bone mineral density (BMD) [17]. Seven days post-injection, the mean corrected calcium level decreased from 9.2 ± 0.5 mg/dL to 8.5 ± 0.5 mg/dL in the treatment group (*P* < 0.01). The calcium level was corrected (mean 9.2 ± 0.9 mg/dL) after 30 days. They did not observe statistically significant iPTH changes: median baseline 164 pg/mL, at 1 week post-injection 224 pg/mL and at 1 month 161 pg/mL. At 1 year, BMD increased by 2.6% ± 4.4% in the denosumab group whereas BMD decreased by 4.5% ± 7.7% in the control group (*P* < 0.001) [17].

Lastly, a recent meta-analysis study by Thongprayoon et al. used six observational studies to determine the incidence of hypocalcemia and the effects on BMD after denosumab use in ESRD patients [6]. The pooled estimated hypocalcemia incident was 42% (95% CI 29%–55%, I2 = 0%) after using denosumab in this special population. Most patients reached their lowest calcium levels in the first 2 weeks up to 2 months after the drug administration. Serum calcium levels did not show significant changes when baseline and post-treatment (≥3 months after treatment) calcium levels were compared. Although symptomatic hypocalcemia requiring hospitalization after denosumab use has been reported in ESRD patients [15], the majority of patients experience asymptomatic hypocalcemia. There was a reduction in PTH levels with standardized mean differences of −1.89 (95% CI −3.44 to −0.34). The authors stated that the decrease in PTH levels may be a result of supplements initiation (calcium and active vitamin D) after initial PTH increase within the first month. Consequently, post-treatment PTH levels were significantly lower than the baseline PTH levels [6]. 

Our patients’ response to denosumab (decrease Ca level and increase iPTH level) was similar to several studies reported in ESRD patients [7,8,10,15,16,17]. The transient surge of iPTH level in Patient 2 is most likely caused by a compensatory mechanism. However, some suggest that denosumab may be decreasing cortical porosity (reducing bone remodeling) by indirectly reversing microarchitectural deterioration which causes transient increases in iPTH levels [14]. Parathyroid hormone (endo- or exo-genous) may also affect osteoblasts by utilizing its osteoanabolic effects [15].

## 4. Conclusions

Hypocalcemia is a common adverse effect of denosumab use in ESRD patients. Intact PTH responses can be drastically different to serum calcium decreases in ESRD patients as presented in this case report. Frequent monitoring of iPTH and Ca levels may be warranted. Perhaps, the best approach for these transient changes for Ca and iPTH may be wait-and-watch, and appropriate supportive care.

## Figures and Tables

**Table 1:** Patient Baseline Characteristics.

	Patient 1	Patient 2
Age (years)	62	72
Gender	Male	Male
Ethnicity	Hispanic	Hispanic
T-score (femoral neck)	−3.3	−4.1
25-hydroxy VitD (ng/mL)	48	41
Serum calcium (mg/dL)	8.8	9.2
iPTH (pg/mL)	264	420
Ca dialysis bath (meq/L)	2.5	2.5
Calcimimetic use	No	No
Dialysis vintage (in years)	2	11
Prior denosumab use	Once	None
Prior bisphosphonate use	Alendronate	No
Phosphate binder use	Calcium carbonate ^+^	Sevelamer carbonate ^#^
Calcitriol use *	Yes	Yes

Abbreviation: Ca = calcium; iPTH = intact parathyroid hormone; VitD = vitamin D. * Patient 1 used 0.25 mcg three times weekly and Patient 2 used 0.25 mcg daily. ^#^ Patient was taking sevelamer carbonate 2.4 g three times daily. ^+^ Patient was taking calcium carbonate 500 mg twice daily.
